# A Fault-Tolerant Localization Method for 5G/INS Based on Variational Bayesian Strong Tracking Fusion Filtering with Multilevel Fault Detection

**DOI:** 10.3390/s25123753

**Published:** 2025-06-16

**Authors:** Zhongliang Deng, Ziyao Ma, Haiming Luo, Jilong Guo, Zidu Tian

**Affiliations:** School of Electronic Engineering, Beijing University of Posts and Telecommunications, Beijing 100876, China; dengzhl@bupt.edu.cn (Z.D.); lhm422@bupt.edu.cn (H.L.); gjl20010323@bupt.edu.cn (J.G.); 741753914@bupt.edu.cn (Z.T.)

**Keywords:** 5G, INS, fusion filtering, fault detection

## Abstract

In this paper, for the needs of high-precision and high-continuity localization in complex environments, a modeling method based on time-varying noise and outlier noise is proposed, and variational Bayesian strong tracking filtering is used for 5G/INS fusion localization. A hierarchical progressive fault detection mechanism is proposed to detect IMU rationality faults and consistency faults in 5G observation information. The main contributions are reflected in the following two aspects: first, by innovatively introducing Pearson VII-type distribution for noise modeling, dynamically adjusting the tail thickness characteristics of the probability density function through its shape parameter, and effectively capturing the distribution law of extreme values in the observation data. Afterward, this article combined the variational Bayesian strong tracking filtering algorithm to construct a robust state estimation framework, significantly improving the localization accuracy and continuity in non-Gaussian noise environments. Second, a hierarchical progressive fault detection mechanism is designed. A wavelet fault detection method based on a hierarchical voting mechanism is adopted for IMU data to extract the abrupt features of the observed data and quickly identify faults. In addition, a dual-channel consistency detection model with dynamic fault-tolerant management was constructed. Sudden and gradual faults were quickly detected through a dual-channel pre-check, and then, the fault source was identified through AIME. Based on the fault source detection results, corresponding compensation mechanisms were adopted to achieve robust continuous localization.

## 1. Introduction

In recent years, the navigation and positioning system has evolved from basic space information technology to the core infrastructure of the national security strategy [1], and at the same time, people have a wide range of application needs in material production and daily life, which has a pivotal impact on the promotion of modern socio-economic development. As an important support for the modern digital society, its application value in the field of civil–military integration has been growing exponentially, not only providing precise spatial and temporal benchmarks for the modernization of national defense, but also deeply empowering key areas such as intelligent traffic management, spacecraft orbit control, and environmental dynamic monitoring, which significantly improves the operational efficiency of socio-economic activities. However, a single navigation system is limited by the signal coverage blind area, multipath effect interference, and long-term stability, as well as other inherent defects. It is difficult to meet the demand for high-reliability full-scene location services. Based on the fusion of multi-source information, navigation technology research has become an inevitable choice to break through the bottleneck of technology.

The fifth-generation mobile communication system, as a new generation of communication technology, has realized a wide distribution on a global scale. In many application scenarios oriented towards smart cities, smart industries, smart agriculture, and so on [2], more intelligent terminals need to be accessed and controlled through the network to ultimately meet the needs of IoT communication. However, the data collected by massive sensors must be based on their location information to empower industries to enhance their intrinsic value. Supported by location-based services, 5G meets the needs of IoT communications with its low latency, high bandwidth, and wide coverage, and 5G also has the potential to be a key technology for solving GNSS signaling limitations [3]. In the Third Generation Partnership Project (3GPP) Rel-16 release, 5G positioning capability is specified to achieve an accuracy of 3 m (80%) indoors and 10 m (80%) outdoors, with an end-to-end latency of less than 1 s [4]. Commonly, in the implementation of wireless localization using 5G, Papp et al. [5] proposed a deep fusion of time difference of arrival (TDOA) algorithms and 5G small cell networks to design a low-complexity and highly scalable indoor localization system framework that reduces the effects of multipath effects and clock bias on TDOA measurements. To be independent of base station synchronization, Menta E Y et al. [6] quantified the nonlinear effect of base station density on the angle of arrival (AOA) positioning performance in 5G ultra-dense networks (UDNs) and verified the practical feasibility and accuracy optimization potential of AOA localization for high-density scenarios through ray-tracing simulations. According to their results, the probability of a sub-meter 2D error is 95%. However, it is worth noting that 5G solutions are overly dependent upon a network foundation of standardization, large bandwidth, low latency, and wide coverage, and 5G positioning still faces technical challenges, such as heterogeneous network synchronization errors and dynamic interference suppression.

On the other hand, the inertial navigation system is a typical paradigm for autonomous navigation [7], which utilizes gyroscopes and accelerometers solidly attached to the carrier to measure the angular and linear motion parameters of the carrier, and then solves the carrier’s navigation information, such as velocity, position, and three-dimensional attitude, through the integration operation, which has many advantages, such as high sampling frequency, low noise, strong stealth, no external interference, and good short-term stability, etc. But, due to the fact that the acceleration and angular velocity will be integrated once and twice during the positioning solution, the system positioning error that exists will accumulate with the integration process, and the long-term working accuracy is poor. In the process of positioning solving, primary and secondary integration operations will be performed on the acceleration and angular velocity, in which the system positioning error will be accumulated with the integration process, and the long-term working accuracy is poor. The advantages and disadvantages of 5G and an inertial navigation system are complementary, so 5G+IMU is a continuous and reliable navigation solution that can provide both long-term and short-term accuracy.

IMU and 5G can be coupled using different coupling methods, including loose, tight, and deep coupling [8]. Filtering algorithms for fusing measurements from several different systems reduce the redundancy and contradiction of the information between sensors to estimate certain state vectors in real-time. The Kalman filter is one of the most-used integration algorithms. The Kalman filter algorithm proposed by R. E. Kalman in 1960 [9] uses the minimum variance estimation as the optimal estimation criterion. The algorithm is a powerful, widely used, and theoretically complete algorithm for linear and discrete systems that can estimate the past, current, and future states of a signal. The extended Kalman filter (EKF) algorithm [10] was then created to solve nonlinear problems in most real-world systems, and the extended Kalman filter (EKF) was proposed, which linearizes the nonlinear dynamics using a first-order approximation of the Taylor series expansion and partial derivatives computed using the Jacobi matrix. The unscented Kalman filter (UKF) [11] uses the probability distribution of the untraceable transform approximation of the nonlinear system as the core mechanism without the need to compute the Jacobi matrix, which significantly reduces the algorithmic complexity and the loss of accuracy introduced by the linearization and achieves highly accurate and computationally inexpensive state estimation. The federal Kalman filter algorithm proposed by Calson [12] adopts the principle of information distribution to obtain different federal Kalman filter (FKF) structures, as well as the corresponding different fault-tolerance, accuracy, and computational size. The variance upper bound technique is used to make the sub-filter time updating and the measurement updating independently, and to obtain the global optimum from local filtering to global filtering. The fusion algorithm from local filtering to global filtering is simple, less computationally intensive, and fault-tolerant. The cubature Kalman filter (CKF) algorithm proposed by Arasaratnam and Haykin, based on the basic principle of Kalman filtering [13], uses the principle of numerical integration of volumes to approximate the mean and covariance of the state of a nonlinear system with additional Gaussian noise by using a set of volume points using a third-order spherical radial volume criterion. As for covariance, the algorithm has made great progress in terms of accuracy and stability. However, the outlier noise and time-varying noise are serious in complex scenes, and the above method cannot solve the above problems well. The variational Bayesian cubature Kalman filter (VBCKF) [14] simplifies the a posteriori probability computation by introducing the variational Bayesian method, and it can adjust the variance of the measurement noise, which provides a solution to the time-varying noise problem. Although the fusion filtering architecture of 5G/INS has made good progress in positioning accuracy, further research is still needed on complex environments under time-varying noise and outlier noise.

In a 5G/INS fusion localization system, not only does fusion filtering ensure the effectiveness of localization, but it also needs to detect the faults of the localization system to determine the availability of the system to make a quick response. Existing fault detection methods, such as the chi-square detection method, the sequential probability ratio test (SPRT), and the autonomous integrity monitoring extrapolation (AIME) method, have certain limitations. The cardinality detection method is effective in detecting hard faults, but the detection of soft faults is not sensitive enough, and there is often a delay phenomenon [15]. The SPRT method is sensitive in detecting both hard and soft faults, but it is prone to false alarms after the end of the faults [16]. The AIME method has a very high accuracy in detecting faults, but it is complicated to compute. And the real-time performance is poor. In response to these shortcomings, some scholars have long proposed the idea of combining the two methods for co-detection. Ren [17] proposed a single-component co-detection of each component of the residuals using residual chi-square detection and SPRT detection to adapt to the system’s ability to track the dynamic characteristics. Compared with the single detection method, the single-component joint detection improves the sensitivity of the detection, but the improvement is limited. Yang [18] designed a comprehensive fault detection scheme of residual cardinality combined with improved SPRT with a moving detection cycle, but the code of this scheme will be more cumbersome. And there are too many judgment conditions leading to inefficiency. Wag [19] proposed a fast reset SPRT method. When the fault disappears and returns to the normal state, the fault is detected by the cardinality test. When the fault disappears back to the normal state, it responds to the sudden disappearance of the fault through the auxiliary judgment of the chi-square test and clears the accumulated detection function to zero. This method is effective, but the subsequent detection function may still receive the influence of the previous samples.

Aiming at the above problems, this paper makes the following contributions:

(1) In this paper, the noise is modeled using a Pearson VII-type distribution, which has a flexible tail control capability and a multi-parameter adjustment mechanism, can adjust the thickness of the tail of the probability density function through the shape parameter, and capture the phenomenon of the frequent occurrence of extreme values in the observed data. The 5G/INS tightly coupled model is localized with high accuracy and continuity by using variational Bayesian strong tracking filtering based on the above model;
(2) In this paper, a hierarchical fault detection structure is proposed, which adopts the rationality detection based on the combination of hierarchical voting mechanism and wavelet analysis to screen the boundary of the observation data output from the inertial components, so as to deal with the abrupt change phenomenon in time, and adopts the consistency of two-channel detection of dynamic fault tolerance management to focus on the temporal correlation, which ensures the continuity and convergence of the evolutionary trajectory of the navigation data in the neighboring cycles and improves the positioning system’s robustness. After fault identification, this paper proposes a robust fault proposal and compensation scheme to reduce the impact of faults on the localization system.

## 2. 5G/INS Fusion Positioning System Model

INS and 5G fusion localization generally has three models: loose coupling, tight coupling, and deep coupling. Loose coupling takes 5G and INS as two independent localization systems, respectively, and fuses them by simple weighting or a filtering method in the localization solving result layer. In actual engineering, loose coupling has strong compatibility, and if a single module fails, it can be repaired and replaced individually, which has less impact on the whole system. But the information utilization rate is low, and it cannot effectively utilize the observation information of 5G and INS. And it requires high performance from the solving algorithm. Different from loose coupling, deep coupling deals with the signal layer data of 5G and inertial sensors, deeply integrates the two systems, makes full use of the useful information of both, has strong anti-interference ability, and effectively improves the reliability of the positioning system, but it still has the problems of poor scalability and compatibility and is difficult to implement in software and hardware. Combining the characteristics of the above two fusion models, this section of the paper adopts a tightly coupled model for the fusion of 5G and INS, which directly processes the volume layer information and is still able to estimate the localization results through the inertial information of high frequency when the quality of the 5G signals is poor, and has a high degree of feasibility.

### 2.1. 5G/INS Tightly Coupled Localization Equation of State

In the tightly coupled positioning system of 5G and INS, the design of the state equations needs to consider the dynamic characteristics of 5G and the inertial system at the same time. Since the INS system has a higher dynamic response and update frequency, the state matrix constructed in this paper is dominated by the INS and is assisted by using the 5G positioning system to correct the cumulative error of the INS that increases gradually with time.

The inertial navigation error model in the ECEF coordinate system can be expressed by the continuous type 5G+INS error state equation as:

(1) $$\left.\left\{\begin{array}{c} \delta {\dot{r}}_{eb}^{e}=\delta \nu_{eb}^{e}\\ \delta {\dot{\nu }}_{eb}^{e}=R_{b}^{e}f^{b}\times \phi_{be}^{e}+R_{b}^{e}\delta f^{b}-2\omega_{ie}^{e}\times \delta \nu_{eb}^{e}+\delta g^{e}\\ {\dot{\phi }}_{be}^{e}=-R_{b}^{e}\delta \omega_{ib}^{b}-\left(\omega_{ie}^{e}\times \right)\phi_{be}^{e}\\ \delta {\dot{b}}_{g}=-\frac{1}{\tau_{b_{g}}}\delta b_{g}+w_{g}\\ \delta {\dot{b}}_{a}=-\frac{1}{\tau_{b_{a}}}\delta b_{a}+w_{a} \end{array}\right.\right.$$

where $\delta b_{g}$ and $\delta b_{a}$ denote the zero-bias errors of the gyroscope and accelerometer, respectively, and $\delta g^{e}$ denotes the deviation between the accelerometer and the actual gravitational acceleration, which contains $\delta b_{a}$. $w_{g}$ and $w_{a}$ denote the driving white noise of the gyroscope and the accelerometer, respectively, and $\tau_{b_{g}}$ and $\tau_{b_{a}}$ denote the correlation of the first-order Gauss–Markov processes of the gyroscope’s and accelerometer’s respective times. If the time of arrival (TOA) of 5G is used as the observation, there is a receiver clock difference. To eliminate it, TDOA is used as the distance observation information of 5G in this section. Therefore, the error state vector of 5G+INS tight coupling can be expressed as:

(2) $$\delta x={\left[\begin{array}{ccccc} (\delta r_{eb}^{e}{)}^{T} & (\delta v_{eb}^{e}{)}^{T} & (\phi_{be}^{e}{)}^{T} & \delta b_{a}^{T} & \delta b_{g}^{T} \end{array}\right]}^{T}$$

The error state transfer equation for the continuous type can be expressed as:

(3) $$\delta \dot{x}(t)=F(t)\delta x(t)+\omega (t)$$

where F(t) denotes the state transfer matrix of the continuous-time system, and ω(t) denotes the system noise vector. In practical engineering, discrete data need to be processed, and the above continuous-type state transfer matrix can be discretized as:

(4) $$x_{k}=F_{k,k-1}x_{k-1}+w_{k-1}$$

(5) $$F_{k,k-1}=exp[\int_{t_{k-1}}^{t_{k}}F(t)dt]\approx exp\left\{F(t_{k-1})\Delta t\right\}\approx I+F(t_{k})\Delta t$$

where I is the unit vector, Δt denotes the time interval between neighboring INS observation calendar elements, and $F_{k,k-1}$ can be expressed as:

(6) $$F_{k,k-1}=\left[\begin{array}{ccccc} I_{3} & I_{3}\Delta t & 0_{3} & 0_{3} & 0_{3}\\ N^{e}\Delta t & I_{3}-\left[2\omega_{ie}^{e}\times \right]\Delta t & \left[R_{b}^{e}f^{b}\times \right]\Delta t & R_{b}^{e}\Delta t & 0_{3}\\ 0_{3} & 0_{3} & I_{3}-\left[\omega_{ie}^{e}\times \right]\Delta t & 0_{3} & -R_{b}^{e}\Delta t\\ 0_{3} & 0_{3} & 0_{3} & \frac{\tau_{b_{g}}-\Delta t}{\tau_{b_{g}}}I_{3} & 0_{3}\\ 0_{3} & 0_{3} & 0_{3} & 0_{3} & \frac{\tau_{b_{a}}-\Delta t}{\tau_{b_{a}}}I_{3} \end{array}\right]$$

where $I_{3}$ denotes a 3×3 unit matrix, and $0_{3}$ denotes a 3×3 all-0 matrix.

The process noise matrix is:

(7) $$Q=diag(0_{3},I_{3}\sigma_{a}^{2}\Delta t,I_{3}\sigma_{g}^{2}\Delta t,I_{3}\sigma_{ba}^{2}\Delta t,I_{3}\sigma_{bg}^{2}\Delta t)$$

where $\sigma_{a}$ denotes the random wandering of the accelerometer, $\sigma_{g}$ denotes the random wandering of the gyroscope, $\sigma_{ba}$ is used to denote the zero-value offset instability of the accelerometer, and $\sigma_{bg}$ is used to denote the zero-value offset instability of the gyroscope.

### 2.2. 5G/INS Fusion Positioning Measurement Equation

The 5G/INS tightly coupled positioning system updates the state matrix in real time through the measurement equation, and when obtaining the observation information of the next calendar element, the observation vector is obtained by finding the difference between the measured value of the wireless signal and the virtual result of the INS projection. The observation value of the 5G positioning system includes the distance and the angle, the distance is generally expressed by the TDOA, and the angle is classified into the directional angle Azi and the pitch angle Pit. The observation equation of the 5G/INS tightly coupled system can be expressed as follows. The observation equation of the tightly coupled system can be expressed as:

(8) $$\begin{array}{c} Z_{k}=\left[\begin{array}{c} {TDOA}_{2,k}^{5G}-{TDOA}_{2,k}^{INS}\\ \vdots \\ {TDOA}_{i,k}^{5G}-TDOA_{i,k}^{INS}\\ {Azi}_{1,k}^{5G}-{Azi}_{1,k}^{INS}\\ \vdots \\ {Azi}_{i,k}^{5G}-{Azi}_{i,k}^{INS}\\ {Pit}_{1,k}^{5G}-{Pit}_{1,k}^{INS}\\ \vdots \\ {Pit}_{i,k}^{5G}-{Pit}_{i,k}^{INS} \end{array}\right]=H_{k}X_{k}+V_{k} \end{array}$$

where $Z_{k}$ denotes the 5G/INS tightly coupled error observation vector; ${TDOA}_{i,k}^{5G}$, ${Azi}_{i,k}^{5G}$, and ${Pit}_{i,k}^{5G}$ denote the TDOA, direction angle, and pitch angle observation information of the $i^{th}$ base station at the moment of k, respectively; $TDOA_{i,k}^{INS}$, ${Azi}_{i,k}^{INS}$, and ${Pit}_{i,k}^{INS}$ denote the TDOA, direction angle, and pitch angle information of the *i*th base station derived from INS at the k time, respectively.

The coordinates obtained based on the 5G positioning algorithm are usually the position information of the phase center of the terminal’s 5G antenna, while the coordinates obtained through the mechanical arrangement of the inertial navigation system are usually the measurement center of the IMU. The two cannot be mounted to the same point in the actual terminal, and the positional deviation caused by the difference between the two measurement centers mentioned above is known as the pole-arm error. In static no-rotation scenarios, the rod-arm error can be ignored when the demand for positioning accuracy is not high, but in chemical scenarios, where the staff operates frequently and needs to obtain fine position information, it must be compensated.

Assuming that the position vector of the phase center of the 5G antenna under the e-system is denoted as $r_{5G}^{e}$, and the position vector of the measurement center of the INS under the e-system can be denoted as $r_{INS}^{e}$, the rod-arm error can be expressed as:

(9) $$r_{5G}^{e}=r_{INS}^{e}+R_{b}^{e}l_{INS,5G}^{INS}$$

where $l_{INS,5G}^{INS}$ denotes the pole-arm vector, which is the projection in the b-system of the vector from the measurement center of the INS to the phase center of the 5G antenna.

The distance from the phase center of the 5G antenna to the *i*th base station projected from INS is:

(10) $${TOA}_{i}^{INS}=\left|{base}_{i}^{e}-r_{5G}^{e}\right|+w_{clc}=\left|{base}_{i}^{e}-r_{INS}^{e}-R_{b}^{e}l_{INS,5G}^{INS}\right|+w_{clc}$$

(11) $${TDOA}_{j}^{INS}={TOA}_{j}^{INS}-{TOA}_{1}^{INS}$$

where ${base}_{i}^{e}$ denotes the coordinates of the *i*th base station in e system, ${TOA}_{i}$ is the distance measurement from the *i*th base station to the terminal, i=1,2,…,N, N is the total number of measuring base stations, $w_{clc}$ denotes the clock difference of the terminal, and ${TDOA}_{j}$ denotes the time of arrival difference between the *j*th base station and the first one, j=2,…,N. It can be seen that TDOA can eliminate the interference of the receiver’s clock difference in ranging.

To facilitate the subsequent arithmetic, let

(12) $${∆r}_{i}={base}_{i}^{e}-r_{5G}^{e}$$

The angle information measured by each base station can be obtained by ${∆r}_{i}$:

(13) $${Azi}_{i}^{INS}=arctan\left(\frac{{∆r}_{i,y}}{{∆r}_{i,x}}\right)$$

(14) $${Pit}_{i}^{INS}=arctan\left(\frac{{∆r}_{i,z}}{\sqrt{{{∆r}_{i,x}}^{2}+{{∆r}_{i,y}}^{2}}}\right)$$

The observation transfer matrix can be constructed from the above information:

(15) $$H_{t,k}^{5G}=\left[\begin{array}{ccccc} E_{1,k}^{RTT} & 0_{1\times 3} & 0_{1\times 3} & 0_{1\times 3} & 0_{1\times 3}\\ \vdots & \vdots & \vdots & \vdots & \vdots \\ E_{n,k}^{RTT} & 0_{1\times 3} & 0_{1\times 3} & 0_{1\times 3} & 0_{1\times 3}\\ E_{1,k}^{Azi} & 0_{1\times 3} & 0_{1\times 3} & 0_{1\times 3} & 0_{1\times 3}\\ \vdots & \vdots & \vdots & \vdots & \vdots \\ E_{n,k}^{Azi} & 0_{1\times 3} & 0_{1\times 3} & 0_{1\times 3} & 0_{1\times 3}\\ E_{1,k}^{Ele} & 0_{1\times 3} & 0_{1\times 3} & 0_{1\times 3} & 0_{1\times 3}\\ \vdots & \vdots & \vdots & \vdots & \vdots \\ E_{n,k}^{Ele} & 0_{1\times 3} & 0_{1\times 3} & 0_{1\times 3} & 0_{1\times 3} \end{array}\right]$$

where the nonzero elements of the state transfer matrix can be obtained by taking a partial derivative of the observation equation.

(16) $${\left.E_{n,k}^{RTT}=\left[\begin{array}{c} \frac{\partial {TDOA}_{n,k}^{INS}}{\partial x}\\ \frac{\partial {TDOA}_{n,k}^{INS}}{\partial y}\\ \frac{\partial {TDOA}_{n,k}^{INS}}{\partial z} \end{array}\right.\right]}^{T},E_{n,k}^{Azi}={\left[\begin{array}{c} \frac{\partial Az_{n,k}^{INS}}{\partial x}\\ \frac{\partial Az_{n,k}^{INS}}{\partial y}\\ \frac{\partial Az_{n,k}^{INS}}{\partial z} \end{array}\right]}^{T},E_{n,k}^{Pit}={\left[\begin{array}{c} \frac{\partial {Pit}_{n,k}^{INS}}{\partial x}\\ \frac{\partial {Pit}_{n,k}^{INS}}{\partial y}\\ \frac{\partial {Pit}_{n,k}^{INS}}{\partial z} \end{array}\right]}^{T}$$

## 3. Strong Tracking Fusion Filtering Method Based on Variational Bayesian

### 3.1. Variational Bayesian Filtering

A variational Bayesian (VB) filter combines variational inference with Bayesian filtering for real-time estimation and prediction of the implied state in dynamic systems, and solves the difficult problem of non-Gaussian noise in a 5G/INS fusion positioning system, which is difficult to be quantified accurately by variational approximation of the complex a posteriori probability distribution, and realizes efficient inference of the state variables. Different from traditional Bayesian filtering methods such as Kalman filtering and particle filtering, a variational Bayes converts the problem of solving a posteriori distribution into an iterative optimization problem by solving the maximum variational lower bound instead of the computation of the minimum KL dispersion, avoiding the high computational cost of the Monte Carlo method and easing the integration or summation difficulties that are difficult handle in the exact Bayesian inference.

#### 3.1.1. KL Divergence

The core of Bayesian inference lies in the computation of the posterior distribution, and in complex models, the true posterior distribution is usually difficult to compute directly and needs to be solved by approximation. KL divergence, as a kind of asymmetric index measuring the difference between two probability distributions, can quantify the degree of deviation between the true posterior probability density function p(Φ|Z) and the approximated posterior probability density function q(Φ) and intuitively reflect the information loss of the approximated distribution, where Φ denotes the set of parameters in the state vector and Z denotes the set of parameters in the observation vector. The KL divergence KL(q(Φ)|p(Φ|Z)) between the approximate posterior probability density function obtained by inference with respect to the true posterior probability density function is denoted as:

(17) $$KL(q(\Phi )|p(\Phi |Z))=\int q(\Phi )ln\frac{q(\Phi )}{p(\Phi |Z)}d\Phi \geq 0$$

The KL divergence of both gradually becomes smaller when q(Φ) approaches p(Φ|Z), and the KL divergence can be taken to the minimum when and only when the approximate a posteriori probability density function is equal to the true a posteriori probability density function. The core of the VB-based method is to solve the a posteriori probability density function of the parameter set by minimizing KL(q(Φ)|p(Φ|Z)). A transformation of the above equation yields:

(18) $$KL(q(\Phi )|p(\Phi |Z))+\int q(\Phi )ln\frac{p(\Phi ,Z)}{q(\Phi )}d\Phi =lnp(Z)$$

Let:

(19) $$L(q(\Phi ))=\int q(\Phi )ln\frac{p(\Phi ,Z)}{q(\Phi )}d\Phi$$

where ln⁡p(Z) denotes the logarithmic probability density function for the observation set, whose value is usually a constant, and L(q(Φ)) denotes the lower bound function. The process of solving for the minimum of the KL divergence can be equated to the process of maximizing the lower bound function.

#### 3.1.2. Mean Field Theory

In the process of solving the maximum lower bound function L(q(Φ)), the mean field theory needs to be used to transform the high-dimensional complex posterior distribution into the product of multiple low-dimensional independent distributions, so that the variational inference can efficiently deal with models with high-dimensional parameters, hidden variables, or complex dependencies. At this point, the posterior probability density function of the parameter set can be expressed as:

(20) $$q(\Phi )=\prod_{i=1}^{l}q(\phi_{i})$$

In the process of solving the maximum value of the lower bound function, the optimization problem is converted from joint optimization to chunked optimization, where each low-dimensional independent probability density function is updated one by one, avoiding high-dimensional integration operations and matrix inversion operations. VB assumes that the approximate a posteriori probability density function of a certain parameter only is unknown information, and the approximate a posteriori probability density functions of the other parameters are determined based on the current known information and the mean field. Assuming that the complex correlations between the hidden variables are cut off by enforced independence, the a posteriori probability density functions of all parameters are inferred stepwise through continuous iterations until the algorithm converges.

#### 3.1.3. Variational Bayesian Solution Process

Expanding the lower bound function gives:

(21) L(q(Φ))=∫q(Φ)ln⁡p(Φ,Z)dΦ−∫q(Φ)ln⁡q(Φ)dΦ

The front and back terms are obtained by processing them through mean-field theory, respectively:

(22) $$\int q(\Phi )lnp(\Phi ,Z)d\Phi =\int \prod_{i=1}^{l}q(\phi_{i})lnp(\Phi ,Z)d\Phi =\int q(\phi_{i})E^{\Phi^{-i}}[lnp(\Phi ,Z)]d\phi_{i}$$

(23) $$\begin{array}{ll} \int q(\Phi )\ln q(\Phi )d\Phi & =\int \prod_{i=1}^{l}q(\phi_{i})\ln\prod_{i=1}^{l}q(\phi_{i})d\Phi =\int \prod_{i=1}^{l}q(\phi_{i})\sum_{j=1}^{l}\ln q(\phi_{j})d\Phi \\ & =\sum_{j=1}^{l}\int \prod_{i=1}^{l}q(\phi_{i})\ln q(\phi_{j})d\Phi \\ & =\sum_{j=1}^{l}\int q(\phi_{j})\ln q(\phi_{j})(\prod_{i=1,i\neq j}^{l}\int q(\phi_{i})d\phi_{i})d\phi_{j} \end{array}$$

where:

(24) $$E^{\Phi^{-i}}[lnp(\Phi ,Z)]=\int \prod_{j=1,j\neq i}^{l}q(\phi_{j})lnp(\Phi ,Z)d\phi_{j}$$

Since the posterior probability density function of each low-dimensional parameter satisfies $\int q(\phi_{i})d\phi_{i}=1$, i.e.,

(25) $$\begin{array}{ll} \int q(\Phi )\ln p(\Phi ,Z)d\Phi & =\int q(\phi_{i})\ln exp(E^{\Phi^{-i}}[\ln p(\Phi ,Z)])d\phi_{i}\\ & =\int q(\phi_{i})\ln\frac{exp(E^{\Phi^{-i}}[lnp(\Phi ,Z)])}{\int exp(E^{\Phi^{-i}}[lnp(\Phi ,Z)])d\phi_{i}}d\phi_{i}+C \end{array}$$

(26) $$\int q(\Phi )lnq(\Phi )d\Phi =\sum_{j=1}^{l}\int q(\phi_{j})lnq(\phi_{j})d\phi_{j}$$

where C is a constant, and the lower bound function can be converted to:

(27) $$\begin{array}{ll} L(q(\Phi )) & =\int q(\phi_{i})\ln\frac{exp(E^{\Phi^{-i}}[lnp(\Phi ,Z)])}{\int exp(E^{\Phi^{-i}}[lnp(\Phi ,Z)])d\phi_{i}}d\phi_{i}+C-\sum_{j=1}^{l}\int q(\phi_{j})\ln q(\phi_{j})d\phi_{j}\\ & =-\mathrm{KL}(q(\phi_{i})|q^{*}(\phi_{i}))+C-\sum_{j=1,j\neq i}^{l}\int q(\phi_{j})\ln q(\phi_{j})d\phi_{j} \end{array}$$

Since the range of values of KL divergence is non-negative, to maximize the lower bound function should be satisfied:

(28) $$q(\phi_{i})=q^{*}(\phi_{i})$$

The a posteriori probability density function for each parameter can be obtained by using the formula for the lower bound function. The a posteriori probability density function for the set of parameters can be obtained by using the mean field theory:

(29) $$q(\Phi )=\prod_{i=1}^{l}q(\phi_{i})==\prod_{i=1}^{l}q^{*}(\phi_{i})$$

### 3.2. Variational Bayesian Anti-Noise Filtering Method Based on 5G/INS Positioning System

To realize 5G/INS fusion robust positioning, the 5G observation data and INS prediction data need to be filtered, and the traditional Kalman filter is mainly designed for linear systems and usually adopts a Gaussian state-space-based modeling of the measurement noise, which is assumed to obey a Gaussian distribution. The petrochemical scene is complex, and the simple Gaussian distribution cannot accurately describe the noise information. Therefore, the Kalman filter needs to be improved to broaden its filtering capability in non-Gaussian states.

In complex chemical scenes, the noise of observation information can be divided into two categories. One is the normal observation noise obeying a Gaussian distribution, and the other is the outlier noise with a non-Gaussian distribution when the observation data are seriously disturbed. For the normal observation noise, the traditional Kalman filter can filter it out. For the outlier noise, the method based on Student’s t-distribution is usually used to model it, which can characterize the non-Gaussian noise to a certain extent. However, in the dynamic positioning model of personnel in a complex chemical plant area, the observation noise has an outlier, with mutability and unknown probability distribution, and the statistical variability between different types of noise will be neglected if the overall modeling is used. To solve the above problem, it is necessary to model different types of noise independently and differentiate the different types of noise containing outliers in terms of statistical characteristics.

From the previous subsection, the state space modeling equation can be obtained, and the state one-step prediction equation is:

(30) $$x_{k+1|k}=F_{k}x_{k|k}$$

The error covariance matrix equation for one-step prediction of the state can be expressed as:

(31) $$P_{k+1|k}=F_{k}P_{k|k}F_{k}^{T}+Q_{k}$$

In the a posteriori estimation process, the observation equation can be rewritten to consider the effect of outlier noise:

(32) $$\left\{\begin{array}{c} z_{k+1}=H_{k}x_{k+1}+{\tilde{\nu }}_{k+1}\;,\;y_{k+1}=1\\ z_{k+1}=H_{k}x_{k+1}+{\bar{\nu }}_{k+1}\;,\;y_{k+1}=0 \end{array}\right.$$

where $y_{k+1}$ denotes the judgment factor of the 5G/INS fusion positioning system on the observation noise; ${\tilde{\nu }}_{k+1}$ and ${\bar{v}}_{k+1}$ denote the system’s normal observation noise and the outlier noise at the k+1 moment, respectively; $y_{k+1}=1$ denotes that the observation noise is not interfered with by the outlier at the current moment, and $y_{k+1}=0$ denotes that the observation noise is interfered with by the outlier at the current moment. The normal observation noise can be modeled using the traditional Gaussian distribution:

(33) $$p({\tilde{\nu }}_{k+1})=N({\tilde{v}}_{k+1};\theta^{m},R_{k+1})$$

where p(·) denotes the probability density function; N(∙;μ,Σ) denotes a Gaussian distribution with mean μ and variance Σ; and $R_{k+1}\in R^{m\times m}$ denotes the covariance matrix of the normal observation noise.

Considering the normal observation noise and the outlier noise together, the probability density function of the observation noise can be obtained as:

(34) $$p\left(v_{k+1}\right)=Pr(y_{k+1}=1)p({\tilde{\nu }}_{k+1})+Pr(y_{k+1}=0)p({\bar{\nu }}_{k+1})$$

where:

(35) $$Pr\left(y_{k+1}=1\right)+Pr\left(y_{k+1}=0\right)=1$$

where $Pr\left(\cdot \right)$ is denoted as the probability of an event occurring. To ensure the covariance property of the judgment factors, this section of the paper is modeled using a Bernoulli-based beta distribution.

(36) $$p(y_{k+1}\left|u_{k+1}\right)=u_{k+1}^{y_{k+1}}{\left(1-u_{k+1}\right)}^{1-y_{k+1}}$$

(37) $$p(u_{k+1})=B(u_{k+1};e_{k+1},f_{k+1})=\frac{u_{k+1}^{c_{k+1}-1}(1-u_{k+1}{)}^{f_{k+1}-1}}{Beta(e_{k+1},f_{k+1})}$$

where $u_{k+1}$ denotes the auxiliary parameter and obeys a beta distribution, and $B(\cdot ;e_{k+1},f_{k+1})$ denotes the beta distribution with nonnegative degrees-of-freedom parameters as $e_{k+1}$ and $f_{k+1}$.

Due to the interference of outlier noise, which results in severe heavy-tailed features of the observation noise, it is not possible to characterize the covariance matrix of the observation noise using the traditional Gaussian distribution. For this reason, in this paper, the noise is modeled using the Pearson VII-type distribution, which has a flexible tail control capability and a multi-parameter adjustment mechanism and can adjust the thickness of the tail of the probability density function through the shape parameter to capture the phenomenon of the frequency of extreme values in the observed data. The probability density function of the Pearson type VII distribution can be expressed as:

(38) $$p({\bar{\nu }}_{k+1})=\int_{0}^{+\infty }N({\bar{\nu }}_{k+1};\theta^{m},R_{k+1}/\gamma_{k+1})G(\gamma_{k+1};c_{k+1},d_{k+1})d\gamma_{k+1}$$

where $\gamma_{k+1}$ is denoted as the random variable with observation noise covariance, $c_{k+1}$ and $d_{k+1}$ are non-zero degrees of freedom parameters, respectively, and $G(\cdot ;c_{k+1},d_{k+1})$ is denoted as the gamma distribution.

(39) $$p({\bar{\nu }}_{k+1})=\int p({\bar{\nu }}_{k+1}|\gamma_{k+1})p(\gamma_{k+1})d\gamma_{k+1}$$

It can be further obtained that:

(40) $$p(\gamma_{k+1})=G(\gamma_{k+1};c_{k+1},d_{k+1})=\frac{(d_{k+1}{)}^{c_{k+1}}}{\Gamma (c_{k+1})}(\gamma_{k+1}{)}^{c_{k+1}-1}exp\left(-d_{k+1}\gamma_{k+1}\right)$$

(41) $$p({\bar{\nu }}_{k+1}|\gamma_{k+1})=N({\bar{\nu }}_{k+1};\theta^{m},R_{k+1}/\gamma_{k+1})$$

where $\Gamma (c_{k+1})$ is denoted as the gamma distribution.

Variational Bayes has good adaptive properties and can effectively reduce the effect of outlier noise, so it is introduced into the state estimation. The core concept is to approximate the actual posterior distribution through a relatively simple distribution, thus avoiding the complex integration operation involved in solving the real posterior distribution, which makes it more flexible and efficient when dealing with the combined 5G/INS navigation information fusion problem.

When building the variational Bayesian framework, it is necessary to iteratively update the hidden variables together with the state vectors, and the spatial model of the hierarchical state can be built as:

(42) $$\begin{array}{l} p(\gamma_{k+1},y_{k+1},u_{k+1},x_{k+1},z_{1:k+1})\\ =p(z_{k+1}\left|y_{k+1},x_{k+1},\gamma_{k+1},u_{k+1},z_{1:k}\right)p(x_{k+1}\left|y_{k+1},\gamma_{k+1},u_{k+1},z_{1:k}\right)\\ \times p(y_{k+1}\left|\gamma_{k+1},u_{k+1},z_{1:k}\right)p(u_{k+1}\left|\gamma_{k+1},z_{1:k}\right)p(\gamma_{k+1}\left|z_{1:k}\right)p(z_{1:k})\\ =p(z_{k+1}\left|y_{k+1},x_{k+1},\gamma_{k+1}\right)p(x_{k+1}\left|z_{1:k}\right)p(y_{k+1}\left|u_{k+1}\right|)p(u_{k+1})p(\gamma_{k+1})p(z_{1:k}) \end{array}$$

where:

(43) $$p(z_{k+1}|y_{k+1},x_{k+1},\gamma_{k+1})=N(z_{k+1};H_{k+1}x_{k+1},R_{k+1}{)}^{y_{k+1}}N(z_{k+1};H_{k+1}x_{k+1},R_{k+1}/\gamma_{k+1}{)}^{1-y_{k+1}}$$

Assuming that the posterior probability density function $p(x_{k}|z_{1:k})$ of the state vector at moment k can be expressed as $N(x_{k};x_{k|k},P_{k|k})$, it can be introduced that the predicted probability density function $p(x_{k+1}|z_{1:k})$ of the state vector at moment k+1 can be expressed as $N(x_{k+1};x_{k+1|k},P_{k+1|k})$.

In summary, the hierarchical state space model can be visualized as:

(44) $$\begin{array}{l} p(\gamma_{k+1},y_{k+1},u_{k+1},x_{k+1},z_{1,k+1})\\ =p(z_{1:k})N(z_{k+1};H_{k+1}x_{k+1},R_{k+1}{)}^{y_{k+1}}N(z_{k+1};H_{k+1}x_{k+1},R_{k+1}/\gamma_{k+1}{)}^{1-y_{k+1}}\\ \times N(x_{k+1};x_{k+1|k},P_{k+1|k})u_{k+1}^{y_{k+1}}(1-u_{k+1}{)}^{1-y_{k+1}}B(u_{k+1};e_{k+1},f_{k+1})G(\gamma_{k+1};c_{k+1},d_{k+1}) \end{array}$$

In the process of variational inference, it is necessary to maximize the lower bound function of each variational factor by taking turns to optimize it, and the remaining factors are regarded as known fixed values in each optimization. The parameters of the low-dimensional probability density distribution are updated by using the current expectation values of the other factors. To facilitate the calculation, the low-dimensional probability density function is expressed in logarithmic form in this paper. In the *j*th iteration process, $lnq^{j}(x_{k+1})$ can be expressed as:

(45) $$lnq^{j}(x_{k+1})=C_{x_{k+1}}^{j}-0.5tr[D_{k+1}P_{k+1k}^{-1}]-0.5(E^{j-1}[\gamma_{k+1}](1-E^{j-1}[y_{k+1}])\;+E^{j-1}[y_{k+1}])tr[J_{k+1}R_{k+1}^{-1}]$$

(46) $$D_{k+1}=(x_{k+1}-x_{k+1|k})(x_{k+1}-x_{k+1|k}{)}^{T}$$

(47) $$J_{k+1}=(z_{k+1}-H_{k+1}x_{k+1})(z_{k+1}-H_{k+1}x_{k+1}{)}^{T}$$

where the $C_{x_{k+1}}^{j}$ multilayer state space containing no other low-dimensional subparameters of the state vector is a constant. $E^{j}[f(\theta )]$ is denoted as the statistical expectation of the function f(θ). Taking the above equation exponentially can be expressed as:

(48) $$\begin{array}{ll} q^{j}(x_{k+1}) & =C_{k}^{j}N(x_{k+1};x_{k+1|k},P_{k+1|k})\\ & \times N(z_{k+1};H_{k+1}x_{k+1},R_{k+1}/(E^{j-1}[\gamma_{k+1}](1-E^{j-1}[y_{k+1}])+E^{j-1}[y_{k+1}])) \end{array}$$

where $C_{k}^{j}$ is a constant, and the observation noise writing method matrix, Kalman gain, state vector, and process covariance matrix during the *j*th iteration can be updated sequentially as:

(49) $$R_{k+1}^{j}=\frac{R_{k+1}}{\left(E^{j-1}[\gamma_{k+1}](1-E^{j-1}[y_{k+1}])+E^{j-1}[y_{k+1}]\right)}$$

(50) $$K_{k+1}^{j}=P_{k+1|k}H_{k+1}^{T}(H_{k+1}P_{k+1|k}H_{k+1}^{T}+R_{k+1}^{j}{)}^{-1}$$

(51) $$x_{k+1|k+1}^{j}=x_{k+1|k}+K_{k+1}^{j}(z_{k+1}-H_{k+1}x_{k+1|k})$$

(52) $$P_{k+1|k+1}^{j}=P_{k+1|k}-K_{k+1}^{j}H_{k+1}P_{k+1|k}$$

The above equation shows that, if we want to obtain the covariance matrix $R_{k+1}^{j}$ of the observation noise during the *j*th iteration, we need to obtain the specific information of $E^{j-1}[\gamma_{k+1}]$ and $E^{j-1}[y_{k+1}]$, and in the same way as the above method, $lnq^{j}(y_{k+1})$ can be expressed as:

(53) $$\begin{array}{ll} lnq^{j}(y_{k+1}) & =C_{y_{k+1}}^{j}-0.5y_{k+1}(tr[G_{k+1}^{j}R_{k+1}^{-1}]+ln\left|R_{k+1}\right|+mln2\pi -2E^{j-1}[lnu_{k+1}])\\ & -0.5(1-y_{k+1})\times (E^{j-1}[\gamma_{k+1}]tr[G_{k+1}^{j}R_{k+1}^{-1}]+mln2\pi +ln\left|R_{k+1}\right|\\ & -mE^{j-1}[ln\gamma_{k+1}]-2E^{j-1}[ln(1-u_{k+1})]) \end{array}$$

(54) $$G_{k+1}^{j}=H_{k+1}P_{k+1|k+1}^{j}H_{k+1}^{T}+(z_{k+1}-H_{k+1}x_{k+1|k+1}^{j})(z_{k+1}-H_{k+1}x_{k+1|k+1}^{j}{)}^{T}$$

where $C_{y_{k+1}}^{j}$ is a parameter. Since the judgment factor dominates the iterative estimation process, $q^{j}(y_{k+1})$ can be approximated as:

(55) $$q^{j}(y_{k+1})\approx (p^{j}(y_{k+1}=1){)}^{y_{k+1}}(p^{j}(y_{k+1}=0){)}^{1-y_{k+1}}$$

(56) $$p^{j}(y_{k+1}=1)=C_{1,0}^{j}exp\left(E^{j-1}[lnu_{k+1}]-0.5(tr[G_{k+1}^{j}R_{k+1}^{-1}])\right)$$

(57) $$p^{j}(y_{k+1}=0)=C_{1,0}^{j}exp\left(E^{j-1}[ln(1-u_{k+1})]-0.5(E^{j-1}[\gamma_{k+1}]tr[G_{k+1}^{j}R_{k+1}^{-1}])\right)$$

The expected value of the judgment factor can be expressed as:

(58) $$E^{j}[y_{k+1}]=\frac{p^{j}(y_{k+1}=1)}{p^{j}(y_{k+1}=1)+p^{j}(y_{k+1}=0)}$$

$lnq^{j}(u_{k+1})$ can be expressed as:

(59) $$lnq^{j}(u_{k+1})=C_{u_{k+1}}^{j}+(e_{k+1}+E^{j}[y_{k+1}]-1)lnu_{k+1}+(f_{k+1}-E^{j}[y_{k+1}])ln(1-u_{k+1})$$

where $C_{u_{k+1}}^{j}$ is a constant.

(60) $$q^{j}(u_{k+1})=B(u_{k+1};e_{k+1}^{j},f_{k+1}^{j})$$

(61) $$e_{k+1}^{j}=e_{k+1}+E^{j}[y_{k+1}]$$

(62) $$f_{k+1}^{j}=f_{k+1}+1-E^{j}[y_{k+1}]$$

(63) $$E^{j}[lnu_{k+1}]=\Psi (e_{k+1}^{j})-\Psi (e_{k+1}^{j}+f_{k+1}^{j})$$

(64) $$E^{j}[ln(1-u_{k+1})]=\Psi (f_{k+1}^{j})-\Psi (e_{k+1}^{j}+f_{k+1}^{j})$$

where $\Psi (a)=-E+\int_{0}^{1}\frac{1-t^{a-1}}{1-t}dt$ is the inverse gamma function, and E is Euler’s constant.

According to mean field theory, $lnq^{j}(\gamma_{k+1})$ can be expressed as:

(65) $$\begin{array}{c} lnq^{j}(\gamma_{k+1})=C_{\gamma_{k+1}}^{j}+0.5(m(1-E^{j}[y_{k+1}])+2c_{k+1}-2)ln\lambda_{k+1}\\ -0.5(2d_{k+1}+(1-E^{j}[y_{k+1}])tr[G_{k+1}^{j}R_{k+1}^{-1}])\lambda_{k+1} \end{array}$$

where $C_{\gamma_{k+1}}^{j}$ is a constant.

(66) $$q^{j}(\gamma_{k+1})=G(\gamma_{k+1};c_{k+1}^{j},d_{k+1}^{j})$$

(67) $$c_{k+1}^{j}=0.5(m(1-E^{j}[y_{k+1}])+2c_{k+1})$$

(68) $$d_{k+1}^{j}=0.5(2d_{k+1}+(1-E^{j}[y_{k+1}])tr[G_{k+1}^{j}R_{k+1}^{-1}])$$

(69) $$E^{j}[\gamma_{k+1}]=\frac{c_{k+1}^{j}}{d_{k+1}^{j}}$$

### 3.3. Strong Tracking Correction Method Based on Dynamic Asymptotic Factors

In the previous subsection, the effect of non-Gaussian outlier observation noise in complex chemical scenarios is considered, and the default process noise is Gaussian noise. But, the covariance matrix $Q_{k}$ of the state noise in real environments is also uncertain information, which will lead to a large error in the extrapolation of the error covariance matrix $P_{k+1|k}$ in the one-step prediction process. To further improve the 5G/INS positioning system’s ability to cope with sudden changes in state noise, this subsection utilizes the principle of orthogonality in strong tracking filtering to introduce the fading factor, increase the weight of the current moment data, and correct the covariance matrix of one-step prediction to achieve a fast response to sudden changes.

The core idea of strong tracking filtering is to amplify the covariance matrix of the localization system by adaptively adjusting the filtering parameters when there is an abrupt change in the model state and, thus, maintain the fast tracking of the real state, which can be expressed as:

(70) $$P_{k+1|k}={\beta_{k}F}_{k}P_{k|k}F_{k}^{T}+Q_{k}$$

where $\beta_{k}\geq 1$ is denoted as the dynamic asymptotic cancellation factor of the system. When the system state is smooth, $\beta_{k}\approx 1$. The error covariance matrix equations can be updated using traditional methods. When a sudden change in the state is detected, $\beta_{k}>1$, the Kalman gain is improved by increasing the prediction value of the covariance matrix to respond quickly to new observations.

(71) $$\beta_{k}=max\left(1,\frac{tr(N_{k})}{tr(M_{k})}\right)$$

For the convenience of subsequent operations, the filter residuals are defined as:

(72) $$\epsilon_{k}=z_{k}-H_{k}x_{k|k-1}$$

The covariance $N_{k}$ of the actual residuals can be expressed as:

(73) $$N_{k}=B_{k}-H_{k}Q_{k}H_{k}^{T}-R_{k}^{j}$$

where $B_{k}$ can be expressed as:

(74) $$B_{k}=\left\{\begin{array}{l} \epsilon_{k}\epsilon_{k}^{T},k=1\\ \frac{\eta B_{k-1}+\epsilon_{k}\epsilon_{k}^{T}}{1+\eta },k>1\;\;\;\; \end{array}\right.$$

where $\eta \in \left(0,1\right]$ is denoted as the forgetting factor.

The covariance $M_{k}$ of the theoretical residuals can be expressed as:

(75) $$M_{k}=H_{k}F_{k}P_{k-1|k-1}F_{k}^{T}H_{k}^{T}$$

## 4. Multi-Layer Based Fault Detection Method for 5G/INS

Fault detection based on a 5G/INS localization system requires an intelligent diagnosis of real-time filtered data, the core of which lies in the implementation of a two-dimensional detection of the system’s operating state: rationality detection and consistency detection. Reasonability detection is performed by presetting parameter thresholds to screen the boundary of the observation data output from the inertial components. If an abnormal, abrupt change occurs in the monitoring value, the sensor fault determination mechanism can be triggered, and data isolation can be performed. The consistency verification focuses on the temporal correlation and verifies the continuity and convergence of the evolution trajectory of the navigation data in the adjacent cycles by analyzing the dynamic filtering parameters. When the residual difference between the predicted value of the 5G/INS fusion filter and the actual observed value breaks through the confidence interval, it indicates that there is a risk of system-level deviation.

### 4.1. IMU Observation Information Rationality Fault Detection Method Based on the Combination of Hierarchical Voting Mechanism and Wavelet Analysis

In the inertial navigation system, the random noise of gyroscopes and accelerometers is one of the main factors affecting navigation performance, and it is difficult to establish an accurate error model due to the characteristics of this noise, such as non-stationarity, weak nonlinearity, and slow time-varying. In addition, small errors will accumulate over time, leading to significant deviations in attitude solving and position projection. The zero-bias error of the gyroscope will trigger the attitude angle to diverge at quadratic speed under the integration effect, and the high-frequency noise of the accelerometer may evolve into violent oscillations in the position computation after the quadratic integration. In this regard, this subsection employs a wavelet fault detection method based on a hierarchical voting mechanism to preprocess the inertial sensor observation data before 5G/INS fusion filtering. The wavelet fault detection method does not need to establish a systematic error model and has the ability to multi-scale decomposition the data, which can accurately find the high-frequency detail coefficients dominated by the noise information and perform adaptive threshold shrinkage, which not only suppresses the interference of the sensor noise but also avoids the problem of the traditional low-pass filter that has a poor differentiation between the real signals and the noise, resulting in the loss of useful information.

**Figure 1:**
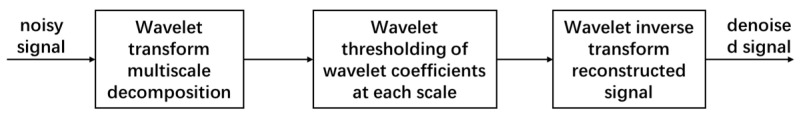
Wavelet denoising flowchart.

The wavelet-thresholding noise reduction technique is a denoising method based on wavelet transform, which is divided into three parts: multi-scale decomposition, thresholding, and signal reconstruction, as shown in Figure 1.

In the process of multi-scale decomposition of signals, the wavelet transform can characterize the local features of signals in two domains of time and frequency and usually decomposes signals into two molecular bands of low-frequency, high-scaled approximation parameters and high-frequency, low-scaled detail parameters. The noise is usually concentrated in the high-frequency details, and the effective signals are usually significant in the low-frequency or specific high-frequency performances.

**Figure 2:**
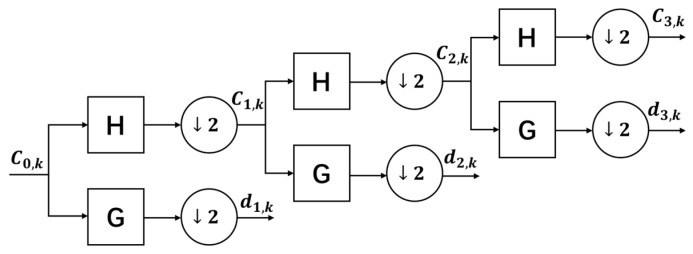
Schematic diagram of wavelet decomposition.

The output information of both gyroscopes and accelerometers are discrete values. Therefore, this subsection discusses the wavelet transform of the discrete signal $f(x_{n})$, which can be expressed as:

(76) $$WT_{f}(j,k)=2^{-j/2}\sum_{n=0}^{N-1}f(x_{n})\psi (2^{-j}n-k)$$

where $WT_{f}(j,k)$ are the wavelet coefficients. The computational complexity is high when using the above equation directly, and ψ(x) usually cannot be expressed using an explicit formula. So the wavelet transform is realized recursively using the two-scale equation

(77) $$\left\{\begin{array}{c} c_{j,k}=\sum_{n}h_{n-2k}c_{j-1,n}\\ d_{j,k}=\sum_{n}g_{n-2k}c_{j-1,n} \end{array}\right.$$

where $h_{k}$ denotes the low-pass filter corresponding to the scale function, and $g_{k}$ denotes the high-pass filter corresponding to the wavelet function. The above process of wavelet decomposition can be visualized from the filter perspective, as shown in Figure 2.

Where H denotes the low-pass filter, G denotes the high-pass filter, and “↓2” denotes the extraction method of discrete signals, i.e., sampling every interval of one point. The original signal is decomposed by the first layer of the wavelet to obtain the corresponding high-frequency signal and low-frequency signal, and then, the low-frequency signal repeats the same decomposition steps, and so on, to complete the signal decomposition process of all the layers.

After completing the multi-scale signal decomposition, the wavelet coefficients corresponding to the high-frequency signals need to be a thresholder to leave the useful signals and remove the noise information, and this subsection adopts the soft threshold function for detection, which ensures the smoothness of the signal detection, reduces the sensitivity of the threshold function to the noise information, and improves the stability.

After wavelet thresholding for noise reduction, to highlight the characteristics of the fault information, a sliding window is used to calculate the local energy information at each scale:

(78) $$E_{i}[n]=\sum_{k=0}^{L_{i}-1}|d_{i}[n-k]{|}^{2}\cdot w[k]$$

where $d_{i}[n]$ denotes the detail function of the *i*th layer wavelet decomposition after the denoising process, w[k] denotes the dynamic window function, and $L_{i}=2^{i}-1$ denotes the length of the dynamic sliding window, which increases with the increase of wavelet decomposition layers.

The adaptive detection threshold can be obtained by jointly estimating the local energy distribution with the global statistical information:

(79) $$\tau_{i}=\alpha \cdot \sigma_{i}\sqrt{2lnN_{i}}\cdot \sqrt{\frac{1}{N_{i}}\sum_{n=1}^{N_{i}}E_{i}[n]}$$

where $\sigma_{i}=\frac{MAD(d_{i})}{0.6745}$ denotes the noise standard deviation obtained from the median absolute deviation estimation, $N_{i}$ is denoted as the length of the detail coefficients of the *i*th layer, and α denotes the empirical adjustment factor.

After mapping the detection positions of each layer to the original signal coordinate system, the detection results of each layer are synthesized, and the rising edge of inertial sensor faults is detected using a multi-scale joint decision-making method based on a hierarchical voting mechanism:

(80) $$Fault[n]=\left\{\begin{array}{l} 1,\;\;\;\;P[n]\geq K\\ 0,\;\;\;\;P[n]<K\;\;\;\; \end{array}\right.$$

(81) $$P[n]=\sum_{i=1}^{L}\delta_{i}[n]$$

where K denotes the number of layers of the hierarchical voting mechanism, and $\delta_{i}[n]$ denotes as the judgment result of the local energy information and the adaptive threshold, if $E_{i}[n]>\tau_{i}$, then $\delta_{i}\left[n\right]=1$, otherwise, $\delta_{i}\left[n\right]=0$.

### 4.2. Coherent Dual-Channel System Cooperative Fault Detection Method Based on Dynamic Fault Tolerance Management

Reasonable fault detection mainly considers the judgment of the current state but lacks consideration for the temporal continuity of the positioning terminal motion state. Therefore, consistency fault detection is needed. At present, the mainstream consistency fault detection methods include chi-square detection, sequential probability ratio detection, and AIME detection. The chi-square detection method includes the state chi-square detection method and the residual chi-square detection method. The state chi-square detection method can better capture implicit faults in the system model, but it may lose some real-time performance. The residual chi-square detection method can directly reflect abnormal observed values and respond quickly to sudden faults, but it relies on a single accurate model and has a high false alarm rate during dynamic changes. SPRT has a low computational cost, fast detection speed, and can effectively control the false alarm rate and the missed alarm rate. But, it only supports binary decision-making and is not sensitive to composite faults. The chi-square detection method and the SPRT detection method can achieve rapid detection, but neither can classify faults. In the 5G/INS fusion positioning system, the introduction of angle measurement in 5G observation increases the redundancy of calculations. In the fault detection process, it is necessary to judge angle measurement faults or distance measurement faults.

To achieve fast and robust fault detection, this paper adopts the fading SPRT detection and residual chi-square detection methods as the pre-check layer to timely screen suspected fault periods. Then, AIME is used to achieve accurate fault classification through multi-model analysis, realizing the dynamic fault-tolerant management of multiple faults, greatly reducing computing resources, and avoiding continuous high-load operations.

#### 4.2.1. Residual Chi-Square Detection

The residual chi-square detection method is used to quickly identify sudden faults in 5G positioning systems by analyzing the deviation characteristics between the actual observed values and the predicted values.

When no faults occur, the residuals follow a Gaussian distribution with a zero mean, which can be expressed as:

(82) $$S_{k}=H_{k}P_{k,k-1}H_{k}^{T}+R_{k}$$

When there is a system malfunction, the mean of the residuals is no longer zero. The statistical measure of residual chi-square detection can be constructed through the Mahala Nobis distance of residuals:

(83) $$\kappa_{k}=\epsilon_{k}^{T}S_{k}^{-1}\epsilon_{k}$$

Among them, $\kappa_{k}$ is the chi-square distribution with m degrees of freedom, and m is the dimension of the measurement vector. When the system fails, the residual mean deviates from zero, the covariance increases abnormally, the statistics follow a non-central chi-square distribution, and the mean deviates significantly. The rules for fault diagnosis can be expressed as:

(84) $$\left\{\begin{array}{l} \kappa_{k}>T_{D},\;\;\;\;Fault\\ \kappa_{k}\leq T_{D},\;\;\;\;No\;Fault\;\;\;\; \end{array}\right.$$

Among them, $T_{D}$ is the threshold value for fault detection, which can be solved based on the false alarm rate α:

(85) $$P_{f}=\int_{T_{D}}^{\infty }\chi^{2}\left(\kappa ,m\right)d\kappa =1-\int_{0}^{T_{D}}\chi^{2}\left(\kappa ,m\right)d\kappa =\alpha$$

#### 4.2.2. Gradual SPRT Detection

Suppose the *k*th order through independent samples of a certain normally distributed random variable x is denoted as $\left\{\epsilon_{i}|i=1,2,...,k\right\}$, which can be further denoted as $\epsilon ∼N\left({\bar{\epsilon }}_{k},A_{k}\right)$ through the theory of probability statistics:

(86) $$\left\{\begin{array}{l} {\mu =\bar{\epsilon }}_{k}=\frac{1}{k}\sum_{i=1}^{k}\epsilon_{i}\\ {A_{k}=\sigma }_{k}^{2}=\frac{1}{k}\sum_{i=1}^{k}(\epsilon_{i}-{\bar{\epsilon }}_{k}{)}^{2}\;\;\;\; \end{array}\right.$$

Among them, ${\bar{\epsilon }}_{k}$ represents the mean residual of the filter, which can be divided into two situations: with fault and without fault. $A_{k}$ can be expressed as the variance of the filter residuals. Now, for the malfunction, the statistical property of $\epsilon_{k}$ is a Gaussian distribution deviating from the zero mean, and the binary assumption of $\epsilon_{k}$ can be expressed as:

(87) $$\left\{\begin{array}{l} H_{0},No\;Fault\;\;\;\;E[\epsilon_{k}]=0,E[\epsilon_{k}\epsilon_{k}^{T}]=A_{k}\\ H_{1},Fault\;\;\;\;E[\epsilon_{k}]=\mu ,E\{[\epsilon_{k}-\mu ][\epsilon_{k}-\mu {]}^{T}\}=A_{k}\;\;\;\; \end{array}\right.$$

The probability density functions of the two can be expressed as, respectively:

(88) $$p(\epsilon_{k}/H_{0})=\frac{1}{\sqrt{2\pi A_{k}}}exp\left[-\frac{1}{2}\epsilon_{k}^{T}A_{k}^{-1}\epsilon_{k}\right]$$

(89) $$p(\epsilon_{k}/H_{1})=\frac{1}{\sqrt{2\pi A_{k}}}exp{\left[-\frac{1}{2}[\epsilon_{k}-\mu \right]}^{T}A_{k}^{-1}[\epsilon_{k}-\mu ]]$$

According to the criterion of maximum a posteriori probability, the likelihood ratio can be expressed as:

(90) $$L_{k}=\frac{p(\epsilon_{1},\epsilon_{2},\ldots \epsilon_{k}|H_{1})}{p(\epsilon_{1},\epsilon_{2},\ldots \epsilon_{k}|H_{0})}=\prod_{j=1}^{k}\frac{p(\epsilon_{j}|H_{1})}{p(\epsilon_{j}|H_{0})}$$

By taking the logarithm of the above equation, the statistical value of SPRT can be obtained:

(91) $$\begin{array}{ll} \lambda_{k} & =\ln\left(L_{k}\right)=\ln\left(\prod_{j=1}^{k}\frac{p(\epsilon_{j}|H_{1})}{p(\epsilon_{j}|H_{0})}\right)\\ & =\ln\left(\prod_{j=1}^{k-1}\frac{p(\epsilon_{j}|H_{1})}{p(\epsilon_{j}|H_{0})}\right)+\ln\left(\frac{p\left(\epsilon_{k}|H_{1}\right)}{p\left(\epsilon_{k}|H_{0}\right)}\right)\\ & =\lambda_{k-1}+\Delta \lambda_{k} \end{array}$$

In the traditional fault diagnosis process, the thresholds $T_{up}$ and $T_{down}$ are determined by the false alarm rate $P_{f}$ and the missed detection rate $P_{m}$.

(92) $$T_{up}=ln(\frac{1-P_{m}}{P_{f}})$$

(93) $$T_{down}=ln(\frac{P_{m}}{1-P_{f}})$$

At this point, the fault discrimination rule can be expressed as:

(94) $$\left\{\begin{array}{l} {Fault：\lambda }_{k}>T_{up}\\ {Detection：T}_{down}\leq \lambda_{k}\leq T_{up}\\ {NoFault：\lambda }_{k}<T_{down}\;\;\;\; \end{array}\right.$$

When the statistic is between the threshold values $T_{up}$ and $T_{down}$, the system will not directly make a fault diagnosis but will continue to monitor until the statistic crosses the threshold. During this period, there will be a lag in fault discrimination, and the system cannot timely and accurately determine whether the current observation information has entered the fusion filtering step, which will affect the real-time positioning performance. In addition, dual threshold design usually aims to reduce false alarm rates, but false alarm rates have a more serious impact on positioning results. A high false alarm rate means that there are faults that have not been isolated in a timely manner, which brings greater errors to the positioning system. Therefore, this section adopts a single threshold for fault discrimination, forcing the output of deterministic fault discrimination results at each moment, eliminating decision delays and improving the real-time performance of the positioning system. Therefore, the fault judgment rule of SPRT can be modified to:

(95) $$\left\{\begin{array}{l} \lambda_{k}>T_{up}\;\;Fault\\ \lambda_{k}\leq T_{up}\;\;No\;Fault\;\;\;\; \end{array}\right.$$

The mean of the residual samples can be expressed iteratively as:

(96) $${\bar{\epsilon }}_{k}=\frac{k-1}{k}{\bar{\epsilon }}_{k-1}+\frac{1}{k}\epsilon_{k}$$

The use of SPRT for fault detection heavily relies on historical information, resulting in delays in identifying the beginning and end of faults. In the initial stage of the fault, ${\bar{\epsilon }}_{k}$ is greatly affected by residual samples from historical fault-free moments, resulting in a slow increase in the statistical rate and a long time to reach the threshold, which can lead to long-term missed alarms. Similarly, after the fault ends, if the fault lasts for a long time, ${\bar{\epsilon }}_{k}$ is greatly affected by the residual samples of historical fault moments, and the statistical decrease rate is slow. It takes a long time to fall below the threshold, which can cause long-term false alarms.

To improve the real-time performance of fault detection and reduce the false alarm and missed alarm rates, this section adopts a fading factor to enhance the sensitivity of fault detection. At this point, the residual calculation formula can be revised to:

(97) $${\bar{\epsilon }}_{k}=\frac{k\beta -1}{k\beta }{\bar{\epsilon }}_{k-1}+\frac{1}{k\beta }\epsilon_{k}$$

Among them, β is the fading factor, which can generally be selected as 0.85, which can weaken the influence of historical observation information on the statistic.

#### 4.2.3. AIME Detection

Assuming that no system failure occurs before the first k−1 moments, then, the state estimation before the kth moment is correct, and the residuals $\epsilon_{k}$ and its covariance matrix $M_{k}$ at the *k*th moment are obtained from Section 3.3, where $\epsilon_{k}$ is a Gaussian random vector with a mean value of zero. When the system failure occurs at that moment, the probability distribution of the residuals $\epsilon_{k}$ is changed, and it can no longer be represented by a Gaussian noise with a mean value of zero. So that the system faults can be detected by the detection of the mean value of $\epsilon_{k}$, the fault of the system can be detected. The fault detection statistic can be constructively represented as:

(98) $$s_{avg}^{2}=(r_{avg}^{T})(V_{avg}^{-1})(r_{avg})$$

where:

(99) $$r_{avg}=(V_{avg}^{-1}{)}^{-1}\sum_{k=1}^{m}V_{k}^{-1}\epsilon_{k}$$

(100) $$V_{avg}^{-1}=\sum_{k=1}^{m}M_{k}^{-1}$$

The test statistic $s_{avg}^{2}$ obeys a central chi-square distribution when the 5G/INS positioning system is functioning normally without failures. When failures occur, $s_{avg}^{2}$ obeys a non-central chi-square distribution. The judgment criterion can be expressed as:

(101) $$\left\{\begin{array}{l} s_{avg}^{2}>T_{r}\;\;\;faults\\ s_{avg}^{2}\leq T_{r}\;\;No\;faults\;\;\;\; \end{array}\right.$$

where $T_{r}$ is denoted as the detection threshold value set in advance.

AIME uses the residual between the measured and predicted values during the filtering process as a state estimation parameter, and its statistical properties directly reflect the consistency between the system state and the measurement. In the fusion positioning system of 5G/INS, Section 4.1 has identified faults in the observation data of inertial sensors through a wavelet detection method based on a hierarchical voting mechanism. On this basis, if there is a fault in the ranging or angle measurement information of a certain base station, the corresponding residual sequence will show cumulative deviation, while the residual information of other normal channels still maintains zero mean characteristics. Based on this difference, the fault information of 5G can be identified and eliminated.

#### 4.2.4. Time-Series Hierarchical Traceability Fault Consistency Detection Method Based on Dynamic Fault-Tolerant Management Mechanism

**Figure 3:**
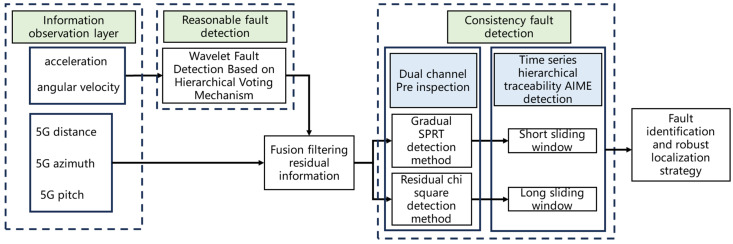
Framework diagram of time-series hierarchical traceability fault consistency detection method based on dynamic fault-tolerant management mechanism.

The core goal of fault detection is to quickly identify and isolate the fault source, ensuring that the system can maintain reliable positioning performance even when some modules fail. To ensure the real-time effectiveness of the 5G/INS fusion positioning system in complex environments, it is necessary to intercept sudden and gradual faults in real time. This paper adopts a temporal hierarchical traceability fault consistency detection method based on a dynamic fault-tolerant management mechanism. The algorithm flowchart is shown in the following Figure 3.

After obtaining the residual information from the fusion filter, a dual-channel pre-check module is constructed using residual chi-square detection and fading SPRT detection methods to quickly identify sudden and gradual faults. The residual chi-square detection method can quickly identify sudden changes in statistics and effectively identify sudden faults, but it is relatively slow in identifying gradual faults. The fading SPRT detection quantifies the confidence of fault occurrence through the logarithmic likelihood function of probability ratio, increases the fading factor to improve the calculation proportion of current residual information, and completes the fastest gradual fault discrimination decision with the least amount of data. Although it can effectively track the system state through iteration, it has a large lag at the end of the fault, and the likelihood ratio takes a long time to recover below the threshold, resulting in an inaccurate judgment of subsequent faults. To ensure the accuracy of multi-fault detection in a time sequence, a SPRT statistic zeroing strategy is added to the pre-check module. Once the residual chi-square detects the end of the fault, the SPRT statistic is zeroed.

The dual-channel layered detection method of the residual chi-square and fading SPRT can quickly identify faults but cannot identify the fault source. Therefore, a temporal layered tracing detection method is introduced after the initial detection module. After obtaining the fault time through initial detection, $N_{window}$ epochs are traced forward for AIME layered detection, where $N_{window}$ is the length of the AIME sliding window. This strategy avoids misjudgment by a single method, reduces the false detection rate of faults, and effectively identifies the fault source by utilizing the cumulative deviation of residual sequences.

AIME uses sliding windows to perform multi-epoch cumulative chi-square tests, improving the sensitivity to cumulative errors in historical data. The length of the sliding window has a significant impact on the detection sensitivity and response time. As the length of the sliding window increases, the AIME statistic increases, and the system’s ability to detect small deviations is improved. But at the same time, the response time is increased. The sensitivity and response time of fault detection are difficult to be compatible, so a reasonable sliding window length should be allocated according to different task requirements. The use of residual chi-square detection can quickly detect sudden faults. If a long window is used for AIME detection, it will cause outliers to scatter across multiple epochs, affecting the sensitivity of detection. Therefore, a short window is required. When detecting gradual faults, deviations accumulate over time. Long windows can fully utilize the temporal information before and after, amplify small deviations, and improve detection capabilities.

#### 4.2.5. Fault Source Identification Method and Fault Repair Strategy Based on AIME

Using the AIME algorithm for fault detection, the normal state, step faults, and slow-change faults correspond to specific models, and the process noise covariance matrix and observation noise covariance matrix of each model can effectively characterize the fault properties. In the normal state, the process noise and observation noise of the system are low. When a step fault occurs, the observation amount suddenly shifts resulting in a sudden increase in the observation noise; when a slow-change fault occurs, the observation amount of the fault corresponds to an increase in the process noise, which not only allows us to determine the type of the fault, but also allows us to determine the specific observation amount of the fault in the 5G system.

The 5G system information layer faults are categorized into three types: only angle faults occur, only distance faults occur, and both angle and distance faults occur.

In this paper, the fourth-order cumulant is used to perform angle measurements under the near-field model [20], which introduces the distance search information after angle measurements without over-increasing the computational complexity, which is dependent upon the accuracy of the angle measurements. Under normal conditions, PRS-based ranging has higher accuracy, and if it fails, only the angle information can be used as the accurate measurement information. The search distance after angle measurement can be introduced instead of the PRS-based measurements, which can provide effective redundant observation information.

Since 5G has single base station localization capability, the fault information can be eliminated when only the distance fails, as well as when both angle and distance fail.

## 5. Experiments and Discussions

This subsection carries out the fault detection of IMU observation information rationality, the fault detection of a 5G/INS fusion localization system, and the verification and analysis of the performance of a 5G/INS tightly coupled fusion filtering algorithm. Since IMU faults and 5G/INS fused localization system faults do not occur frequently in real scenarios. This subsection verifies the validity of the fault detection algorithm by capturing real 5G and IMU observation information, verifying the effectiveness of the fault detection algorithm by artificially adding faults, and verifying and analyzing the performance of the 5G/INS fusion filtering algorithm after rejecting the faults.

### 5.1. Experimental Environment and Parameter Settings

**Figure 4:**
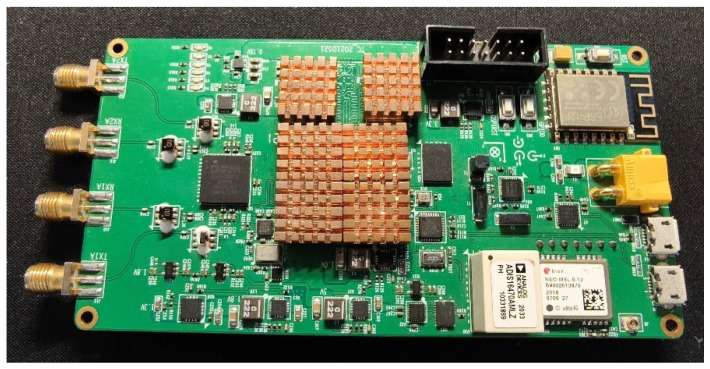
Board for 5G/INS fusion positioning terminal.

In order to verify the fault detection algorithm and the fusion filtering algorithm proposed in this paper, a test environment is set up on the campus of the Beijing University of Posts and Telecommunications (BUPT) Campus, where the localization terminal is fixed on a trolley with a height of 1 m moving at a speed of 0.8–1.5 m/s, and the terminal is equipped with a 5G and inertial guidance module, as shown in the following Figure 4.

The parameter settings for the 5G and inertial sensors are shown in Table 1.

To obtain high positioning accuracy and angular measurement performance, the 5G signal adopts a large bandwidth of 100 MHz, the number of fast Fourier-transform points adopted is 4096, and the base station measurement adopts a 4 × 4 antenna for angular measurement.

### 5.2. Test Results and Performance Analysis

#### 5.2.1. Validation of Fault Detection Methods for Rationalization of IMU Observation Information

**Figure 5:**
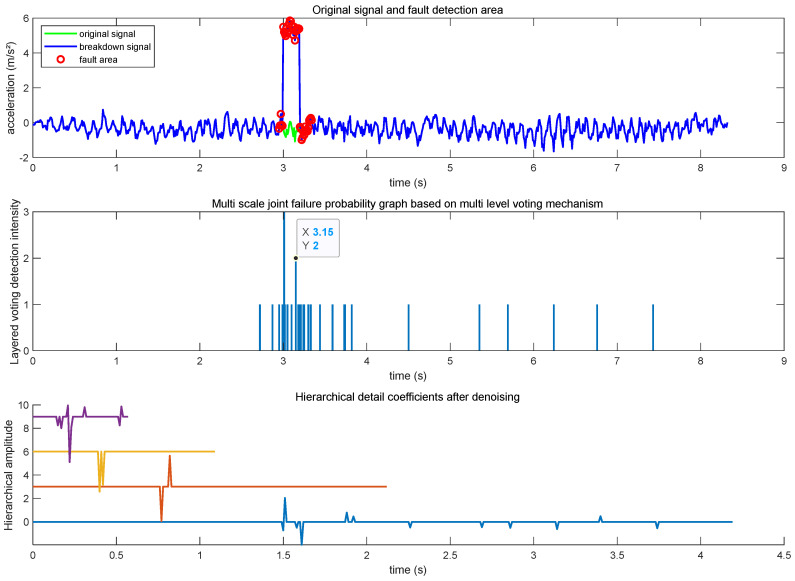
IMU observation information rationalizes fault detection results.

The 8.33s inertial sensor observations are collected on the campus of the Beijing University of Posts and Telecommunications (BUPT), and the fault information is artificially added in the third step in this experiment due to the absence of faults in the normal state. As the smaller bias will be compensated in the 5G/INS fusion filtering process, to reflect the seriousness of the fault information on the positioning system, a larger bias is used to simulate the fault, taking the *y*-axis acceleration observation as an example, and increasing the acceleration bias of 5 m/s^2^ with a duration of 0.2 s. Through the IMU observation fault detection method based on the combination of a hierarchical voting mechanism and the wavelet analysis proposed in this paper, faults can be effectively detected. The information rationality fault detection method can be effective in the detection of faults. To capture the signal’s abrupt change and high-frequency detail information more keenly, this experiment adopts the wavelet base of the fourth order, the number of decomposition layers is four layers, the detection intensity threshold of hierarchical voting is set to two, and the experimental results are shown in the following Figure 5.

The green signal in the first subfigure indicates the original *y*-axis acceleration observation. The blue signal indicates the *y*-axis acceleration observation that has been artificially increased by the fault, and the red signal indicates that a fault is detected in the IMU at that moment. Subfigure 2 indicates the fault detection intensity after hierarchical polling, and subfigure 3 indicates the signal of the four hierarchical scales that have been wavelet-decomposed. A detection intensity threshold of two for hierarchical voting implies that the abrupt change fault exhibits anomalous features on at least two scales at the same time, whereas noisy information or transient interference tends to show anomalous features on a single layer only. From the above figure, the rising and falling edges of the fault can be effectively detected at 3 s and 3.2 s, and an accurate judgment of the fault state can also be made at 0.2 s, during which the abrupt change is maintained.

#### 5.2.2. Validation of a Cooperative Fault Detection Method for Coherent Dual-Channel Systems with Dynamic Fault Tolerance Management

**Figure 6:**
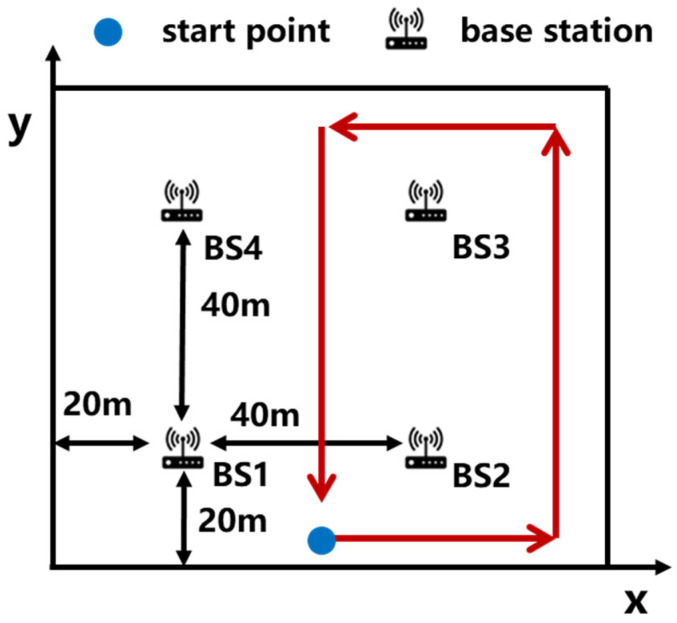
Experimental scene diagram.

To verify the consistency fault detection performance of the 5G/INS fusion positioning system, a testing system was built on the campus of the Beijing University of Posts and Telecommunications, with four base stations, all of which have ranging and angle measurement functions. Establish a relative coordinate system as shown in the following Figure 6, with 40 m between adjacent base stations. The blue circle is the starting point. The red line is the walking route, and the direction of the arrow is the walking direction. The measurement time is 150 s, and the output frequency of the inertial sensor’s observation information is 100 Hz. The output frequency of the 5G observation information is 10 Hz.

To verify the detection ability of the algorithm proposed in this article for sudden and gradual faults, a sudden fault of 10 m was added to the distance measurement observation of base station 2 at the 20th second, with a duration of 3 s. A gradual fault was added to the pitch angle and direction angle of base station 3 at the 100th second, with a duration of 5 s. The fault setting information is shown in Table 2.

**Figure 7:**
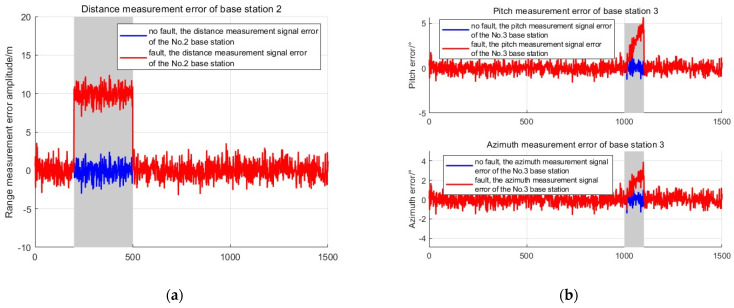
Fault information diagram. (**a**) Base station 2 experienced a sudden malfunction fault; (**b**) base station 3 experienced a gradual change fault.

The fault source generates fault information as shown in the following Figure 7, where the gray area indicates that there is a fault in the measurement information of the base station during that time.

**Figure 8:**
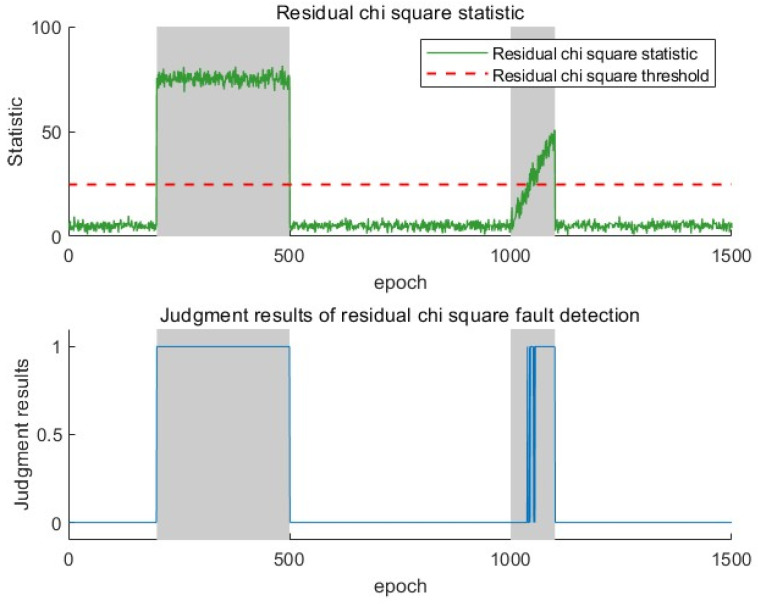
Residual chi-square fault detection statistics and judgment results.

Due to the availability of angle information in all four base stations, this experiment has four elevation angles, four directional angles, and the TDOA observations. Therefore, the degree of freedom m in the chi-square distribution is 11, and the false alarm rate is set to 0.01. The threshold for the residual chi-square detection method can be calculated to be 24.73. In the fading SPRT detection algorithm, if the false alarm rate and missed detection rate are both set to 0.01, the threshold for the SPRT detection method is 4.58. In Figure 8, the gray area indicates the presence of a fault, and the red dashed line represents the threshold of different fault detection methods. A fault detection result of one indicates the occurrence of a fault, while zero indicates the absence of a fault.

The residual chi-square detection method shows significant performance differences in dealing with gradual and sudden faults in Figure 8. The residual chi-square detection method has excellent response characteristics when dealing with sudden faults and can achieve fast response at both the beginning and disappearance stages of the fault. For the initial stage of gradual faults, due to the small measurement deviation, the statistical measures obtained by the residual analysis-based chi-square detection method always remain below a low threshold, making it difficult to capture fault characteristics in a timely manner. The fault signal must be accumulated for a certain period to achieve fault detection. In addition, in the early stages of detecting faults, abnormal observation values will penetrate the measurement update process of the filter, causing the filter output to follow the fault observation values, resulting in frequent switching between the fault state and the normal state, leading to missed alarms.

**Figure 9:**
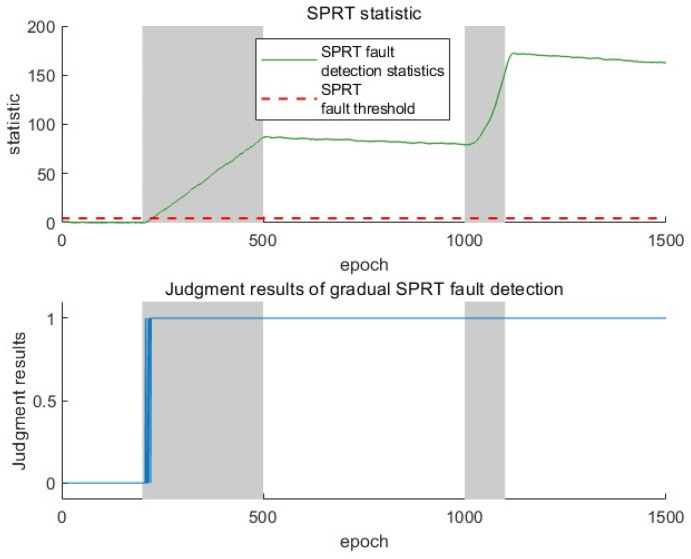
SPRT fault detection statistics and judgment results.

The SPRT-based method performs a fault performance analysis through sequential probability ratios. In Figure 9, when facing sudden faults, it takes several epochs of accumulation to reach the threshold, resulting in a longer response time. When facing gradual faults, it can relatively quickly detect the starting point of the fault. However, after the fault is over, it is impossible to reset the statistic to zero, which means it cannot detect multiple faults.

**Figure 10:**
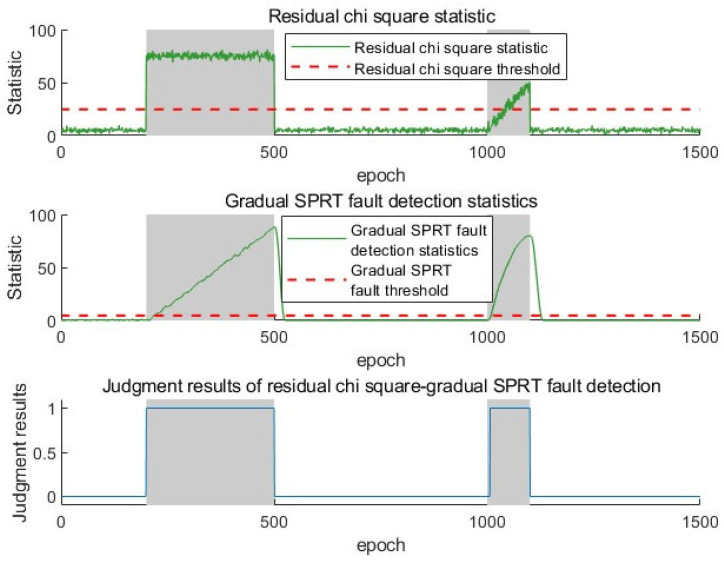
Residual chi-square fading SPRT fault detection statistics and judgment results.

To solve the problems of slow response to gradual faults in residual chi-square detection, insensitivity to fault termination, and inability to detect multiple faults in SPRT detection, the residual chi-square fading SPRT dual channel fault detection algorithm proposed in this chapter is adopted. From the above Figure 10, introducing the fading factor and zeroing strategy can make SPRT more sensitive to fault termination and improve its ability to detect multiple faults. The specific detection results are shown in Table 3.

According to the results in the table above, the combination of a fading SPRT and a statistical zeroing strategy can solve the problem where SPRT can only detect single faults and can also improve the recognition sensitivity at the end of the fault. The residual chi-square fading SPRT dual channel pre-check method can quickly and accurately detect the temporal information of faults but cannot identify the fault source. After pre-check, the AIME method is used for fault detection to avoid misjudgment by a single method, reduce the false detection rate of faults, and accurately identify the fault source using the accumulated deviation of the residual sequence.

**Figure 11:**
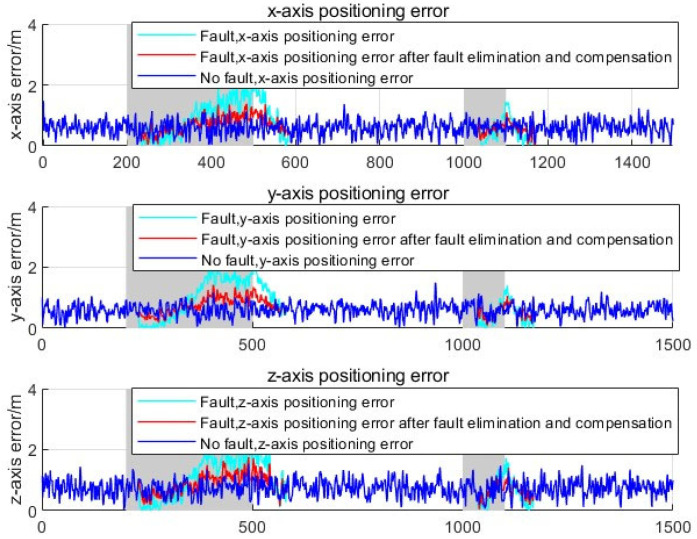
Comparison of positioning errors before and after fault removal.

To visually demonstrate the impact of faults on positioning performance, as well as the improvement of positioning performance through fault detection and repair strategies, this section analyzes the positioning errors on the *x*, *y*, and *z* axes during the positioning process in Figure 11. Since the variational Bayesian strong tracking filter based on Pearson VII distribution proposed in this chapter can suppress time-varying noise and outlier noise, to eliminate the influence of this filtering algorithm on fault detection, a common Kalman filter is used to tightly couple the observation information of 5G and INS. From the below figure, faults have a significant impact on localization performance. By adopting the fault repair strategy proposed in this chapter, when angle information faults are identified, they are eliminated. When a fault is detected in the distance measurement information, the distance measurement information obtained based on the fourth-order cumulative measurement under the near-field model is used as observation data compensation, which can reduce the impact of fault localization performance.

**Figure 12:**
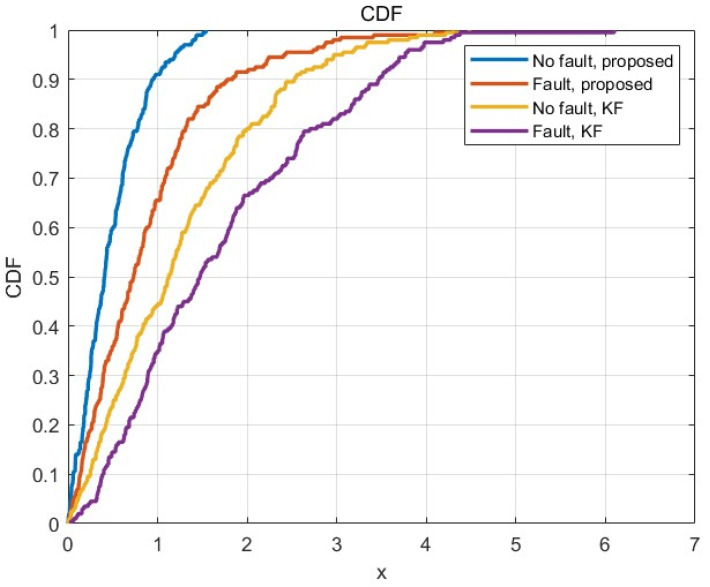
Comparison of fault-free and fault elimination positioning performance.

In addition, this section also compares the fault-free and fault rejection localization performance between the variational Bayesian strong tracking filter based on Pearson VII distribution and the traditional Kalman filter model in Figure 12. The 5G/INS fusion filtering method proposed in this chapter has better performance, and the fault repair strategy proposed in this chapter can reduce the impact of faults on the localization performance and improve the robustness of the 5G/INS fusion positioning system.

#### 5.2.3. Validation of Strong Tracking Filtering Methods Based on Variational Bayesian

**Figure 13:**
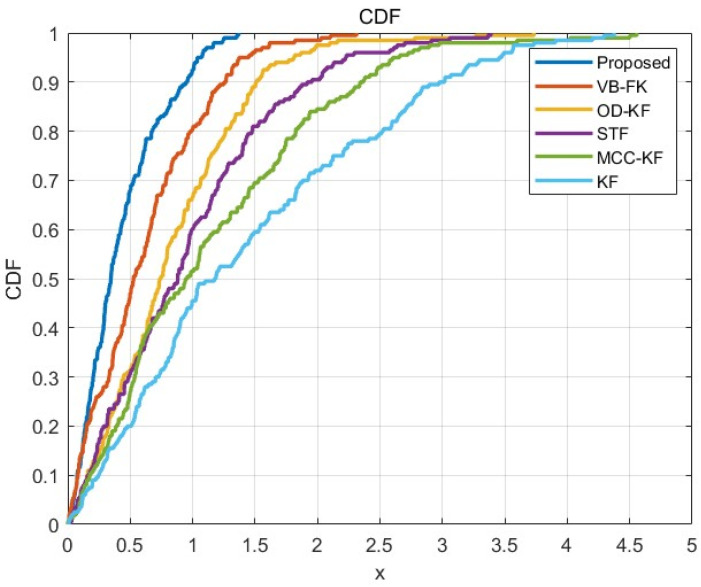
Comparison of localization performance of different filtering algorithms.

To verify the performance of the proposed algorithms in this paper, traditional Kalman filter (KF) [9], maximum correlation entropy Kalman filter (MCCKF) [21], Student’s t-filter (STF) [22], outlier detection Kalman filter (OD-KF) [23], and variational Bayesian Kalman filter (VB-KF) [24] are introduced as the comparison experiments. Among them, the kernel size of the MCCKF is set to σ=100. The degree of freedom parameter in the STF is set to ν=3. The iteration number and the degree of freedom parameter in the OD-KF are set to N=4, $e_{0}=10,f_{0}=0.1$, respectively, and the conventional degree-of-freedom parameter of the variational split Bayesian strong tracking filter proposed in this paper is set as $c_{k+1}=0.01,d_{k+1}=1.01,e_{k+1}=10,f_{k+1}=0.1$, and the number of iterations is set to N=4. The corresponding CDF plots of the localization results are shown below in Figure 13, and the algorithm proposed in this paper has a better localization accuracy and realizes the robust localization with high accuracy of the 5G/INS fusion.

## 6. Summarize

This paper first introduces the basics of inertial navigation systems, and details the common coordinate systems, the parameterization of inertial sensor attitudes, the inertial sensor error modeling, the mechanical orchestration, and the error navigation equations involved in the 5G/INS fusion positioning system. Aiming at the difficult problem that the observation information in complex chemical scenarios is subject to severe outlier interference, leading to the inaccuracy of traditional filters, this paper adopts the Pearson VII-type distribution to model the noise, adjusts the thickness of the tail of the probability density function through the flexible tail control capability and multi-parameter tuning mechanism, captures the phenomenon of the frequent occurrence of extreme values in the observation data, and proposes a robust filter based on the strong tracking of the different variational Bayesian classes of noise, which are modeled independently, and different classes of noise containing outliers are distinguished in terms of statistical features. In response to the difficulty of the 5G/INS fusion positioning system in complex chemical scenarios, which has faults that lead to a sharp drop in positioning performance, this paper adopts the rationality detection based on the combination of hierarchical voting mechanism and wavelet analysis to screen the boundary of the observation data output from the inertial components, so that the abrupt change phenomena can be dealt with timely, as well as adopts the consistency of the two-channel detection of the focusing temporal correlation of the dynamic fault-tolerance management to ensure the continuity and continuity of the evolution trajectory of the navigation data in the neighboring cycles. The continuity and convergence of the data evolution trajectory enhance the robustness of the localization system.

## Figures and Tables

**Table 1:** Parameter configuration of 5G and inertial sensors.

Parameter	Value	Parameter	Value
Carrier Frequency	3.5 GHz	5G Bandwidth	100 M
Subcarrier spacing	60 kHz	Goniometric Antenna Array	4 × 4
Fast Fourier Transform Points	4096	Number of resource blocks	272
5G Sampling Frequency	100 Hz	INS Sampling Frequency	100 Hz
Gyro constant drift	0.1(°)/h	Accelerometer Constant Zero Bias	50 μg
Gyroscope random wander	0.01(°)/h	Accelerometer Random Zero Bias	5 μg

**Table 2:** System fault information setting table.

Fault Source	Fault Time	Fault Type	Fault Model
5G distance	20–50 s	sudden fault	10 m
5G pitch	100–110 s	slow change fault	0.5 × (t − 100)
5G azimuth	100–110 s	slow change fault	0.3 × (t − 100)

**Table 3:** Comparison table of fault detection performance.

Fault Detection Method	Sudden Change Fault Begins	Sudden Change Fault Ends	Gradual Change Fault Begins	Gradual Change Fault Ends	Fault Source Identification
Residual chi-square detection	No delay	No delay	Delay 34 epoch	No delay	Unrecognized
SPRT	Delay 8 epoch	Failure to detect the end of the first fault in the sequence and long-term false alarm			Unrecognized
Gradually eliminating SPRT + Zero setting strategy	Delay 8 epoch	Delay 17 epoch	Delay 7 epoch	Delay 19 epoch	Unrecognized
Residual chi-square –gradual SPRT dual channel detection	No delay	No delay	Delay 7 epoch	No delay	Unrecognized
Residual chi-square–gradual SPRT dual channel detection + AIME	No delay	No delay	Delay 7 epoch	No delay	The sudden fault is the distance measurement information of the base station 2, and the gradual fault is the direction angle and elevation angle of base station 3

## Data Availability

The original contributions presented in the study are included in the article; further inquiries can be directed to the corresponding authors.
